# The Use of Whole-Body Vibration, Electrical Stimulation, and Magnetic Stimulation in Muscle Dystrophy Patients: A Scoping Review

**DOI:** 10.7759/cureus.67051

**Published:** 2024-08-17

**Authors:** Aikaterini Venieri, Nejc Sarabon

**Affiliations:** 1 Faculty of Health Sciences, University of Primorska, Koper, SVN; 2 Sports Excellence/1st Department of Orthopedics, National and Kapodistrian University of Athens School of Medicine, Athens, GRC; 3 Department of Human Health, InnoRenew Co, Izola, SVN

**Keywords:** magnetic stimulation, electrical stimulation, whole-body vibration, sensory input, sensory motor integration, muscle dystrophy

## Abstract

The purpose of this scoping review was to report the effects of vibration therapy, electrical stimulation, and transcranial magnetic stimulation on patients with muscle dystrophies. The outcome measures were muscle strength, body composition, balance, and functional mobility of these patients. We used the Preferred Reporting Items for Systematic reviews and Meta-Analyses (PRISMA) guidelines and the Arksey and O’Malley framework. The literature review was conducted on PubMed. We included studies that were written in English, were peer-reviewed, without regard to the publication date, and implemented a form of “vibration therapy” or “electrical stimulation” or “magnetic stimulation” as an intervention program of any duration. Overall, 14 studies were retrieved. Most of the studies applied whole-body vibration (WBV) therapy or electrical stimulation and only one was found that implemented transcranial magnetic stimulation. The interventions were reported but there was a variety in duration or the frequency of the program, as well as in the disease progression of the patients. It seems that WBV, electrical stimulation, and magnetic stimulation have positive outcomes, but these vary depending on the muscle deficits and limitations of the patients with muscle dystrophy. It is recommended that future studies should be conducted in order to determine the ideal prescription of each intervention, so as to be as beneficial as possible.

## Introduction and background

Muscular dystrophies are genetically inherited neuromuscular disorders, caused by mutation of more than 40 different genes, and mainly affect the muscles [1]. There are several different types of muscular dystrophies with a wide range of symptoms [2]. The most common one in childhood is Duchenne muscular dystrophy (DMD), an X-linked recessive inheritance disorder that affects one in 3,500 males [3]. DMD is caused by mutations in the dystrophic gene [4], which leads to progressive muscle weakness [5]. Reduced muscle strength and functional mobility, difficulty in balance and gait, speech and cognitive delay, or behavioral problems are some of the symptoms that these patients might experience [4,6]. In addition, Becker muscle dystrophy affects 7.3 per 100,000 children, while in adults, the most common form is myotonic dystrophy (10.6 per 100,000 people), followed by facioscapulohumeral dystrophy (3 per 100,000 people) [2]. 

An early diagnosis of muscular dystrophies and immediate treatment can eliminate the risk of clinical complications. High levels of creatinine kinase are an indicator of the disease, but the confirmation of the diagnosis is made by gene testing [4]. The gold standard treatment for these patients is corticosteroids, which slow down the progression of the disease, but it is widely known that there are a lot of adverse effects, such as osteoporotic fractures [5]. An interdisciplinary team of experts, such as cardiologists, physiotherapists, speech therapists, and psychologists, should work together for the treatment of muscular dystrophies, with the exception of pharmacological, molecular, and genetic therapies [4]. 

Even though exercise programs do not have the intention to cure, they are widely recommended for maintaining muscle strength in muscle dystrophy patients [7]. Randomized controlled trials have reported that muscular exercises in the right dosage and intensity can maintain or optimize patients’ functioning and have potential benefits on muscle strength and endurance [8]. Nevertheless, healthcare providers should be aware of the right parameters of exercise for muscle dystrophy patients, as several adverse effects have been reported, such as inflammation and the inability of muscles to repair themselves, which lead to opposite outcomes [9].

Vibration therapy is also a treatment proposed for neurorehabilitation, due to its efficacy and safety [10]. Several studies have been conducted that have assessed the effects of vibration therapy on functional mobility, bone and muscle mass, and muscle stiffness [11,12]. Nonetheless, a standardized protocol about the right direction, dosage, and frequency of vibration as well as amplitude has not been defined yet [13]. Researchers suggest that low-intensity vibration can help reduce the disease’s progression, increase bone density, and enhance motor skills, balance, and upper extremity function [11,14,15]. On the other hand, electrical stimulation seems to have beneficial effects on maintaining muscle strength and preventing atrophies, since it enhances the contraction of muscle fibers, without causing fatigue to the stimulated muscle [16]. Finally, transcranial magnetic stimulation has the ability to activate multiple skeletal muscles depending on the frequency that is applied and has been widely used in treating several neuromuscular disorders [17,18]. 

The aim of this scoping review was to report the effects of vibration therapy, electrical stimulation, and transcranial magnetic stimulation on patients with muscle dystrophies. Due to the heterogeneity of the studies, no restrictions were applied to intervention programs (duration, frequency, intensity, and other exercise parameters), while the outcome measures were muscle strength, body composition, balance, and functional mobility of patients. 

## Review

Methods

Review Guidelines and Framework

We utilized the methodological framework for scoping reviews proposed by Arksey and O’Malley [19], as well as the guidelines in the Preferred Reporting Items for Systematic reviews and Meta-Analyses: extension for Scoping Reviews (PRISMA-ScR) [20]. 

Study Selection Criteria

The inclusion and exclusion criteria were defined based on the PICOS search tool according to Table 1 [21].

**Table 1:** Inclusion and exclusion criteria based on the PICOS search tool.

Inclusion Criteria	
Population (P)	Male or female patients with muscular dystrophies of all ages.
Intervention (I)	Whole body vibration (WBV) therapy, local vibration therapy, electrical stimulation, magnetic stimulation, and stimulated biofeedback training intervention programs of any duration.
Comparisons (C)	Control (healthy) groups receiving no stimulation or placebo stimulation, groups that received any type of exercise, and studies with no control groups were also included.
Outcomes (O)	(a) Muscle strength, not limited to the type of testing or body part; (b) bone density; (c) balance; (d) functional mobility outcomes (timed up-and-go test, sit-to-stand tests, stair climbing, etc.); (e) fatigue.
Study Design (S)	All types of studies that applied at least one of the interventions above were included. There was no restriction on the publication date. Finally, all studies were peer-reviewed and in English
Exclusion Criteria	
(a) Zebrafish models	
(b) Animal studies	

Search Strategy

We searched only in the PubMed database. The database was searched without regard to the date of publication until April 2023 and all appropriate studies were included (14 studies). We used the following combination of keywords: (Musc* dystrophy OR Duchenne dystrophy OR progressive neuromuscular disorders) AND (sensory motor integration OR sensory motor stimulation OR sensory input OR sensory stim integration OR sensory stim stimulation OR sensory-motor stimulation OR sensory-motor integration OR sensorymotor stimulation OR sensorymotor integration OR kinesth* input OR kinaesth* input OR propriocept* OR vibr* OR electric* stimulation OR magnetic stimulation). The database search was performed independently by two authors (N.Š. and A.V.). 

Screening of Studies

After the completion of the search, we extracted the studies that met the inclusion criteria, by reading all the titles and abstracts, so as to be sure about meeting the purpose of this review. Screening of the studies was independently conducted by two individuals (N.Š. and A.V.), who came to an agreement. When there was any debate on inclusion or exclusion, discussion with justification was used until the reviewers came to a consensus. 

Data Extraction and Synthesis

From the selected studies, we identified (a) the study design, (b) the authors and year of publication, (c) the demographics of participants, (d) the intervention characteristics, and (e) the outcomes on muscle strength, bone density, balance, functional mobility outcomes (timed up-and-go test, sit-to-stand tests, stair climbing, etc.) and fatigue. In accordance with the Arksey and O’Malley framework [19], the quality of evidence and the risk of bias were not assessed for the above studies, because the purpose of a scoping review is just a preliminary assessment and an overview of a potentially large and diverse body of literature pertaining to a broad topic.

Results

**Figure 1:**
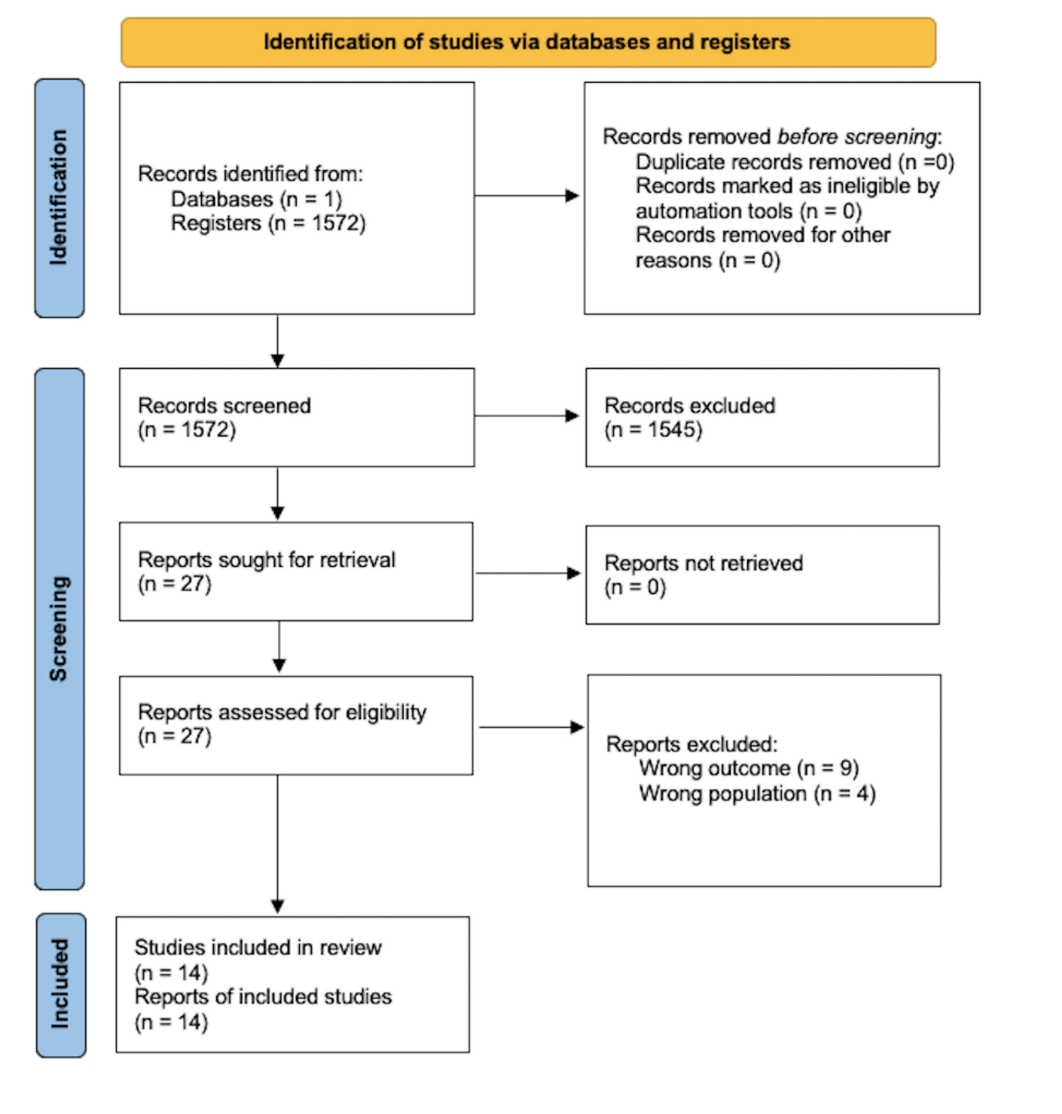
PRISMA flowchart for the scoping review. PRISMA: Preferred Reporting Items for Systematic reviews and Meta-Analyses

A total of 1,572 studies were identified from the PubMed database. After reading all the titles and abstracts, 14 studies met the inclusion criteria and were included in the study (Figure 1).

Summary of Findings

Target population: Table 2 presents all the selected studies and their main characteristics. The sample sizes of the selected studies were quite small, from case studies [14,22] to 24 participants [16,23]. The majority of them included DMD patients [6,12,15,22-26], while the others examined patients with myotonic dystrophy type 1 [18,27], Becker muscle dystrophy [14], and limb-girdle muscular dystrophy [16]. Finally, one compared patients with DMD and those with spinal muscular atrophy [28], and one compared patients with DMD and those with Becker muscle dystrophy [29]. 

**Table 2:** Overview of study characteristics. DMD: Duchenne muscular dystrophy; BMD: Becker muscular dystrophy; WBV: whole-body vibration; MRI: magnetic resonance imaging; DM1: myotonic dystrophy type 1; TENMS: transcutaneous electrical neuromuscular stimulation; EMG: electromyography; RCT: randomized controlled trial

Study	Aim		Participants	Intervention	Results	
Soderpalm et al., 2013 (Prospective observational study) [6].	To study the tolerability of WBV therapy in children with DMD and its effects on muscle and bone.		DMD children (6), 6.8 ± 2 years; Able to walk without assistance; All being treated with 0.35 mg/kg/day of prednisolone.	WBV therapy 12 weeks; 2-3 x/week; 1-2 weeks: 2min/session, 16-24 Hz; 3-12 weeks: 6min/session, 16-18 Hz.	WBV safe and well-tolerated; No significant changes in subjective well-being, bone density, body mass, and muscle strength; No significant change in creatine kinase activity.	
Kurt et al., 2021 (Single-case study) [14].	To study the effects of stimulated biofeedback training on muscle strength, balance, hand function, and transfer activities in a child with BMD and neonatal compartment syndrome.		8-year-old boy with BMD and neonatal compartment syndrome; Balance problems and hand function limitations.	Stimulated biofeedback training Lower extremities: in quadriceps and tibialis anterior muscles; Left upper extremity: supinator muscle; 12 weeks; 3 x/week; 10 min/session.	Statistically significant changes in transfer activities, hand function, grip skills, and in some balance parameters; Minor improvements in hip and knee flexion, extension, and dorsiflexion muscle strength were observed.	
Bell et al., 2017 (Clinical trial – conference abstract) [24].	To investigate the effects of low-magnitude high-frequency vibration and weight-bearing activity compared to a placebo device on bone mineral density in DMD boys.		DMD children (21), 9.3 ± 3.9 years; Ambulant; Received corticosteroids for at least 6 months. Group A: DMD children (11) received WBV therapy. Group B DMD children (10) received vibration with a placebo device.	WBV therapy 12 months; protocol not stated.	Bone mineral density significantly increased after 12 months in the WBV group. All children tolerated the WBV treatment well.	
Bianchi et al., 2022 (Prospective, randomized, double-blind, placebo-controlled clinical trial) [15].	To examine the ability of WBV therapy to prevent skeletal decline in DMD.		DMD children (20); Able to stand and walk; Treated with a fixed dose of prednisone, 25-D supplements, and dietary calcium intake for at least 6 months prior to the study. Group A: DMD boys (10), 9.4 ± 3.1 years; Standing on a WBV device at home; Group B: DMD boys (10), 6.6 ± 1.6 years; Standing on a placebo device.	WBV therapy device at home 12 months; 7 x/week; 10 min/day, 30 Hz. Placebo device at home 12 months; 7 x/week; 10 min/day.	All children tolerated the WBV device well; 4 new fractures in the placebo group; Trabecular bone density of tibia rose slightly in the WBV group; No change from baseline in hip bone mineral density and content in the WBV group; WBV therapy can help protect against the disease progression and complications of chronic steroid use; An important limitation of the study was the higher age of the WBV group, which resulted in greater severity of the disease.	
Petryk et al., 2017 (Pilot study) [29].	To evaluate the feasibility and tolerability of WBV therapy and its effects on muscle function and bone density in patients with dystrophinopathies.		DMD and BMD male patients (5), 12.8 ± 5 years; Able to walk more than 75 m unassisted; Independent from ventilatory support in the daytime.	WBV therapy at home 6 months; 7 x/week; 10 min/day, 30-90Hz.	Feasible and tolerable tool; No adverse effects or fractures; No significant change in walking distance; Timed motor function tests remained the same during the intervention, but then there was a progressive deterioration; Measures of upper extremity strength remained either unchanged or became worse over the 6-month intervention period, whereas measures of lower extremity strength were either stable or showed increased force after the 6-month intervention phase; Motor function and lower extremity muscle strength remained unchanged or improved during the intervention followed by deterioration after the intervention was completed; Bone density and geometry remained stable.	
Myers et al., 2014 (Pilot study) [25].	To examine potential positive effects of WBV therapy on preserving strength, stair climbing, and balance in children with DMD.		DMD patients (4), 10 ± 1.2 years; Ambulant.	WBV therapy 4 weeks; 3 x/week; 4 min/session (2 min on- 1 min interval-2 min on), 7-20 Hz.	Serum creatine kinase was stable; WBV therapy was well-tolerated; No adverse effects; One participant improved in standing, box step climbing, and getting to a sitting position; Some reported subjective improvement in the ankle ROM; Some improvements in single-leg standing and stair climbing.	
Kilinc et al., 2015 (Controlled clinical trial) [16].	To examine the effects of electrical stimulation and exercise therapy on muscle strength and daily life activities in patients with limb-girdle muscular dystrophy.		Limb-girdle muscular dystrophy patients (24); Able to walk without assistive devices; Manual muscle testing 3+ or over. Group A: Electrical stimulation group (11), 31.62 ± 16.92 years. Group B: Exercise therapy group (13), 30.14 ± 11.04 years.	Electrical stimulation 8 weeks; 3 x/week; 10 min/session (pulsing stimulation on deltoideus and quadriceps femoris), 50 Hz. Exercise therapy 8 weeks; 3 x/week; Moderate intensity resistive exercises on deltoideus and quadriceps femoris bilaterally.	Between-group analysis: muscle strength of the Deltoideus was higher in the electrical stimulation group, but the muscle strength of quadriceps was similar in both groups; Within-group analysis: muscle strength of the deltoideus and quadriceps femoris increased bilaterally after treatment in the electrical stimulation group, but only the deltoideus muscle strength increased in the exercise therapy group; Activities of daily living scores did not improve significantly after treatments.	
So et al., 2018 (A case report) [22].	To detect muscle responses to nerve stimulation during peripheral nerve block in 2 children with DMD.		DMD patients (2); aged 2 and 14 years.	Electrical nerve stimulation to the right brachial plexus under general anesthesia. Electrical nerve stimulation to the left femoral nerve.	Muscle responses to nerve stimulation seem to vary according to the severity and progression of the disease.	
Vry et al., 2013 (Clinical trial) [28].	To evaluate the safety of WBV therapy and its effects on muscle strength and ankle range of motion in children with DMD and spinal muscular atrophy.		Group A: DMD children (11); 8.8 years; Able to walk at least 10 m unaided and able to perform specific exercises on the platform. Group B: Spinal muscular atrophy (8), 9.9 years; Able to walk at least 10 m unaided and able to perform specific exercises on the platform.	WBV exercise at home Mild squatting, stretching the gastrocnemius muscle, alternating slight weight shift from the right to the left leg; 8 weeks; 5 x/week, twice/day; 1-4 weeks: 3 min/exercise, 3 exercises/session, 15-18 Hz; 5-8 week: 3 min/exercise, 3 exercises/session, 18-24 Hz.	Good clinical tolerance; DMD group: creatinine kinase increased significantly after 1^st^ day but returned to baseline after 4 weeks of training; small but not significant improvement in their 6-min-walk test and the 4-stairs climbing test; Spinal muscular atrophy group: no statistically significant changes in creatinine kinase; only the increase in the distance walked in 6 min after 8 weeks was statistically significant; Mild but not significant increase in muscle strength and range of dorsiflexion in both groups.	
Scott et al., 1986 (Experimental study) [26].	To examine the effects of chronic low-frequency stimulation on the tibialis anterior muscle of children with DMD.		DMD boys (6), 7.6 ± 2.76 years.	Focal unilateral stimulation on tibialis anterior muscle 7-11 weeks; 3 x/day; 1 hour/session, 5-10Hz.	The stimulated muscles showed a small, but significant increase in strength; There was no significant change in maximum voluntary contraction of the stimulated muscles of the older boys, whereas there was a significant increase in maximum voluntary contraction in younger boys; There was no change in fatigue after chronic low-frequency stimulation.	
Chisari et al., 2013 (Pilot study) [27].		To evaluate the effects of chronic electrical stimulation on walking and muscle strength in DM1 patients.	DM1 patients (6), 42.2 ± 11.5 years.	Electrical stimulation of the tibialis anterior muscle at home 15 days; 2 x/day; 60 min/session, 35Hz.		Increase of muscle strength in patients with mild strength deficit; All subjects improved in the 10-m walking test; 5 patients improved in the 6-min walking test and 4 of them in time up and go test; All reported subjective improvement in walking; Creatinine kinase levels remained the same.
Lacourpaille et al., 2017 (Cohort study) [12].	To assess the effects of DMD on muscle stiffness and response to electrically induced muscle contraction in elbow flexion.		Group A: DMD children (10), 13.6 ± 3.7 years. Group B: Healthy age-matched male controls (9).	Electrically induced contractions on biceps brachii; 2 visits, 1 year apart.	Progressive lengthening of the time required for the muscle force to be transmitted to the skeleton in patients with DMD while maximally evoked torque was unchanged; Increase in muscle stiffness in some muscles at 12 months in children with DMD.	
Trost et al., 2019 (Cross-sectional study) [23].	To evaluate a protocol combining voluntary and evoked muscle contractions to measure strength and excitability of wrist extensor muscles between males with DMD and controls.		Group A: DMD patients (10), 5.4 ± 5.9 years; Able to obtain a neutral wrist extension and perform a 3-sec contraction of the wrist extensor muscles. Group B: Healthy male control group (14), 15.5 ± 5 years; Could not have a neuromuscular diagnosis or any upper extremity mobility limitations.	3 maximum voluntary and 3 stimulated contractions of wrist extensor muscles; 2 visits, 90 days apart.	The procedure was feasible and safe; Reliability and validity were excellent for evoked and voluntary measurements of torque and EMG in DMD patients; Twitch contraction time did not differ significantly between groups, but was able to discriminate patients with DMD.	
Greene et al., 2021 (Concept pilot study) [18].	To test if noninvasive transcranial magnetic stimulation of the primary motor cortex with a new portable wearable multifocal stimulator causes improvement in muscle function in DM1 patients.		DM1 patients (6),45.8 ± 11.8 years; Age between 20-60 years; No changes in medication for 6 months.	Repetitive unilateral active stimulation along with contralateral sham stimulation. 2 weeks; 7 days/week; 40 min/session, 0.2 Hz.	Tolerated the treatment well; No significant changes in muscle function post the intervention; Minor but not significant increase in muscle strength between groups.	

Types of Study

Overall, five pilot studies [18,25-27,29], four clinical trials (one of those was RCT) [15,16,24,28], two cohort studies [6,12], two single-case studies [14,22] and one case-control study [23] were found.

Interventions

There was heterogeneity among studies in the interventions and the protocol they performed. In total, six WBV studies, seven electrical stimulation studies, and one transcranial magnetic stimulation study were found. Among the WBV studies, three reported that participants received WBV at home without supervision [15,28,29] for a period from two to twelve months. In the rest of the studies, only pre-WBV vs. post-WBV changes were observed [6,25] or WBV pre-post changes were compared to sham WBV pre-post changes [24]. The protocol of the interventions was stated in most of the studies. The frequency ranged from seven to 90 Hz and the duration was from two to ten minutes per session. In one study, the protocol of the intervention was not stated, because it was a poster [24]. The weekly frequency was from two to seven days and one session per day, except for one study that performed two sessions per day for five days per week [28]. Vry et al. (2013) compared children with DMD and those with spinal muscular atrophy performing mild squatting, stretching the gastrocnemius muscle, and alternating slight weight shifts from right to left leg on the WBV platform at home (three minutes for each exercise), for four weeks at 15-18 Hz and then at 18-24 Hz for four more weeks [28]. In all the other studies, vibration was applied without performing exercises [28]. 

Among the electrical stimulation studies, only one compared electrical stimulation with an exercise group [16]. Kilinc et al. (2015) applied pulsing stimulation on deltoid muscle and quadriceps femoris in limb-girdle dystrophy patients, for eight weeks, three times per week and 10 minutes per session, while the other group performed moderate intensity resistive exercise on the same muscles bilaterally [16]. Two of the studies used focal stimulation of the tibialis anterior muscle, but the protocols were different [26,27]. Scott et al. (1986) applied focal stimulation unilaterally, in DMD boys, for seven to 11 weeks, three times per day, for one hour, at 5-10 Hz [26], while Chisari et al. (2013) applied stimulation in myotonic dystrophy type I patients only for 15 days, twice a day, for one hour at 35 Hz [27]. Two of the studies compared DMD patients to healthy controls in only two visits. Lacourpaille et al. (2017) performed electrically induced contractions on biceps brachii with visits one year apart [12]. On the other hand, Trost et al. (2019) used three maximum voluntary and three stimulated contractions of wrist extensor muscles 90 days apart [23]. Kurt et al. (2021) applied stimulated biofeedback training to both lower extremities (quadriceps and tibialis anterior muscle) and the left upper extremity (supinator muscle) in one BMD patient [14]. The intervention program lasted for 12 weeks and was held three times per week for 10 minutes per session [14]. Finally, So et al. (2018) performed electrical nerve stimulation once in two DMD patients in order to detect changes in the muscle responses between the subjects. They concluded that muscle responses seem to vary according to the severity and progression of the disease [22]. 

Only one of the included studies applied unilateral active transcranial magnetic stimulation along with contralateral sham stimulation to myotonic dystrophy type I patients. The duration of the intervention was two weeks, and it was implemented daily for 40 minutes per session at 0.2 Hz [18]. 

Effects of WBV, Electrical Stimulation, and Magnetic Stimulation on Muscle Parameters

Most of the studies stated that WBV was safe and well-tolerated by the patients and no adverse effects were reported. Soderpalm et al. (2013) concluded that after applying WBV to DMD patients for three months, bone mass and body composition showed no significant changes, though there was an increasing trend in bone density after three months, but without any statistical significance [6]. Similar effects were found by Bianchi et al. (2022), who used WBV at participants’ homes for 12 months. Even though all children tolerated the treatment well, there were no changes in hip bone mineral density and content from baseline, and trabecular bone density of the tibia rose slightly in the WBV group, but four new fractures were obtained in the placebo group. This is an indication that WBV therapy can help protect against the disease progression and complications of chronic steroid use. An important limitation of the study was the higher age of the WBV group, which resulted in greater severity of the disease [15]. Petryk et al. (2017) concluded that after six months of WBV at home, there were no adverse effects or new fractures, bone density, and geometry remained stable, while upper extremity strength remained either unchanged or grew worse over the intervention period, and measures of lower extremity strength were either stable or showed increased force after the six-month intervention phase [29]. Finally, in an older study, Bianchi et al. (2013) stated that bone mineral density significantly increased after 12 months of WBV, and all children tolerated the treatment well [30].

The results were similar in the studies that used electrical stimulation. Kilinc et al. (2015) compared the use of electrical stimulation or exercise in limb-girdle dystrophy patients and reported that muscle strength of the deltoideus was higher in the electrical stimulation group, but the muscle strength of quadriceps was similar. They found that muscle strength of the deltoideus and quadriceps femoris increased bilaterally in the electrical stimulation group, whereas in the exercise group improvement was found only in the deltoideus muscle [16]. In a case study, So et al. (2018), concluded that muscle responses to nerve stimulation seem to vary according to the severity and progression of the disease, while Scott et al. (1986) also reported that after focal stimulation on tibialis anterior in DMD boys, there was no significant change in maximum voluntary contraction in the older boys, but there was a significant increase in maximum voluntary contraction in the younger boys [22,26]. Similar effects were obtained by Chisari et al. (2013), who performed electrical stimulation on the tibialis anterior muscle in muscle dystrophy type I patients, and reported that patients with a mild strength deficit had an increase in muscle strength [27]. Kurt et al. (2021) performed stimulated biofeedback training in the lower and upper extremities on a BMD patient and after 12 weeks minor improvements in muscle strength of hip and knee flexion, extension, and dorsiflexion were observed [14]. Finally, Lacourpaille et al. (2017) and Trost et al. (2019) performed electrically induced contraction on biceps brachii and wrist extensor muscles respectively, in two visits, and both groups of researchers concluded that the evoked torque was unchanged between the visits but the procedure was reliable and valid to discriminate patients with DMD [12,23]. 

As far as transcranial magnetic stimulation is concerned, Greene et al. (2021) performed repetitive unilateral active stimulation along with contralateral sham stimulation for two weeks and found a minor but insignificant increase in muscle strength between groups [18]. 

Effects of WBV, Electrical Stimulation, and Magnetic Stimulation on Balance, Functional Mobility, and Fatigue 

Most of the studies examined the functional effects of WBV, electrical stimulation, and magnetic stimulation. In WBV studies, the results are quite similar. Petryk et al. (2017) reported no significant changes in walking distance and the performance of the timed motor function tests remained the same during the intervention period, but there was a progressive deterioration during the six months after the end of the study [29]. On the other hand, Myers et al. (2014) concluded that one of the four patients improved in standing position, box step climbing, and getting to a sitting position after a four-week WBV therapy, while there were also some minor but statistically insignificant improvements in single-leg standing and stair climbing in all patients. Finally, some of the patients reported subjective improvement in the range of motion of the ankle [25]. In another interventional program with WBV exercises, the DMD group showed a small but insignificant improvement in the performance of the 6-min walk test and the 4-stair climbing test, but the spinal muscular atrophy group had a statistically significant increase in the distance walked in six minutes after eight weeks of the intervention [28]. 

In the electrical stimulation studies, the results were controversial. Kilinc et al. (2015) reported that the activities of daily living scores did not improve significantly after exercise or electrical stimulation treatment [16]. On the other hand, electrical stimulation of the tibialis anterior muscle seems to be beneficial in DM1 patients, because all subjects improved in the 10-meter walking test, five patients improved in the 6-min walking test and four also improved in the time up and go test. All participants reported subjective improvement in walking [27]. Similar effects were obtained by Kurt et al. (2021), who reported statistically significant changes in transfer activities, hand function, grip skills, and some balance parameters after 12 weeks of stimulated biofeedback training [14].

Finally, no significant changes in muscle function were found after a transcranial magnetic stimulation intervention, even though the DM1 patients tolerated the treatment well [18]. 

Only one study examined the effects of electrical stimulation on fatigue. Scott et al. (1986) performed focal stimulation on the tibialis anterior muscle in DMD boys and did not find any change in fatigue after chronic low-frequency stimulation [26]. 

Discussion

Even though muscle dystrophy has been widely studied for a long time, there was a lack of evidence about therapeutic modalities focused on sensory inputs such as WBV and electrical or magnetic stimulation. Researchers have performed different interventional protocols, so it is challenging to compare them and to extract safe conclusions about the efficacy or inefficacy of an intervention.

Most of the studies focused on bone and muscle mass as well as patients’ functional level. Fatigue was measured in only one study and no changes were detected after chronic low-frequency stimulation [26]. The six WBV studies found no major improvements after the intervention, but the patients who received the vibration did not become worse. WBV therapy was well tolerated and no new fractures or adverse effects were reported. Important limitations of the studies were the higher age of one group that received WBV, which resulted in the worse progression of the disease, as well as small sample sizes [6,15,25,29]. WBV therapy can hinder disease progression, complications of the lower extremity weight bearing, and the reduction of bone density due to medication. 

In the seven studies that applied electrical stimulation, improvements were obtained mainly in the muscle strength of the tibialis anterior muscle [26,27], but this was inextricably linked to the severity of the disease. Patients with mild strength deficits had better outcomes. Furthermore, electrical stimulation appeared to lead to statistically significant changes in transfer activities, as well as in some balance parameters if applied both to the quadriceps femoris and the tibialis anterior muscle, whereas no major differences were reported when it was applied only to quadriceps [14,16].

Finally, only one study using transcranial magnetic stimulation on DM1 patients was found, and no significant changes in muscle function were reported clinically after two weeks of the intervention program. EMG showed significant improvement in muscle action on nervous stimulation of the first dorsal interosseus muscle and a similar but insignificant trend in the trapezius muscle, between groups [18].

As a scoping review, several studies have been included with different designs and methods. The quality of these studies was not examined, since it was not in the scope of this review. Each study performed different protocols and the published results were inconsistent. Furthermore, heterogeneity was observed among the studies with respect to the number of participants, their age, the severity of the disease, and the protocol that each study implemented. The severity and the progression of the disease is an important factor that can affect the results. More randomized controlled trials should be performed with larger sample sizes, specific methodology, and interventional protocols. In addition, only one database was utilized for the purposes of the review of literature for the respective scoping review. Limited resources and human power did not permit extending the search to other databases. It is recommended that more databases will be utilized in the future for the review of the literature. Finally, only studies written and published in English were included.

## Conclusions

In this scoping review, WBV, electrical stimulation, and magnetic stimulation appear to be safe, feasible, and well-tolerated therapeutic modalities for patients with muscle dystrophy, with beneficial effects on disease progression. Electrical stimulation seems to have more promising outcomes. Most of the retrieved studies had limitations, and the results should be generalized with caution. Nevertheless, the ideal application of the frequency, amplitude, and duration are some of the parameters that still need to be determined so as to maximize the therapeutic benefits in this population.
